# Timing of dental development in relation to the treatment of maxillary canines: a retrospective register-based study

**DOI:** 10.2340/aos.v84.45203

**Published:** 2025-12-29

**Authors:** Jenni Ristaniemi, Eeva Melaluoto, Jenni Iivari, Paula Pesonen, Raija Lähdesmäki

**Affiliations:** a Oral Development and Orthodontics, Research Unit of Population Health, Faculty of Medicine, University of Oulu, Oulu, Finland; b Infrastructure for Population Studies, Faculty of Medicine, University of Oulu, Oulu, Finland; c Oral and Maxillofacial Department, Medical Research Center Oulu (MRC Oulu), Oulu University Hospital, Oulu, Finland

**Keywords:** dental age, headgear, human, interceptive treatment, mixed dentition

## Abstract

**Objective:**

To describe differences in the dental age of Finnish children with the mixed stage of dentition in relation to the treatment provided for the permanent maxillary canines.

**Material and methods:**

This retrospective register-based study was based on 1,332 cross-sectional dental panoramic tomographs (DPTs) for children with a chronological age of 8.5–10.5 years together with longitudinal information on the eruption and treatment of 1,817 maxillary canines in the same children. The treatments were categorized into early (interceptive treatment and early headgear) and late treatment (orthodontic treatment and treatment for crowding) groups. Dental age was assessed by Demirjian’s dental maturity method and grouped into delayed (≤ –1 year), normal (> –1 and < +1), and advanced (≥ +1 year) relative to children’s chronological age. Results were performed using Pearson’s chi-square test, Fisher’s exact test, and multinominal logistic regression models.

**Results:**

Normal dental age at the time of the DPT was detected most often in the children in all studied treatment groups. Delayed dental age was detected more often in the children in the early treatment group and advanced dental age in the late treatment group (*p* = 0.002). The mean dental age of the girls with early treated canine(s) lagged significantly behind that of the girls in the groups that received no treatment (–0.43 years, *p* = 0.004) or late treatment (–0.45 years, *p* = 0.026). Delayed dental age was detected in 28.1% of the interceptively treated canines, leading to an association between delayed dental age and interceptive treatment (odds ratio 3.99, 95% confidence interval 1.84–8.67).

**Conclusions:**

Association was found between delayed dental age and interceptive treatment of a maxillary canine. Because of variations in dental age within the same age group, the timing of treatment plays a key role in order to achieve early treatment options for children’s erupting maxillary canines.

## Introduction

The permanent maxillary canines can face multiple problems during their long and complicated eruption pattern [1–3], and in many cases, only orthodontic intervention can ensure their eruption into the dental arch and thus establish its functional position in the occlusion. In addition, the treatment of maxillary canine eruption disturbances is essential for reducing the risk of complications such as root resorption in the adjacent teeth [4, 5].

The timing of dental development is always individual, and there can be wide variation in the eruption schedules of teeth. Dental development and maturation usually take place earlier in girls [6–8], especially at the late mixed stage of the dentition [9], when the maxillary canines erupt into the oral cavity, approximately at the age of 10–12 years [10], but with a deviation of several years [6].

Early diagnosis of maxillary canine eruption disturbances will enable properly timed intervention by means of a number of interceptive treatment options, the aim of which will be to guide the aberrant development of the canine early enough to normalize its eruption path or at least make later orthodontic treatment simpler. It is well known that properly timed extraction of a primary canine is an effective interceptive treatment option when compensating for a palatally displaced permanent canine [11–13], since systematic use of this treatment combined with a precise follow-up of the ectopically erupting maxillary canines has been shown to reduce the prevalence of actual maxillary canine impactions [14].

Since the permanent maxillary canines are among the latest teeth to erupt into the dental arch, their eruption is greatly affected by the space available in the arch. Viewed from this perspective, a lack of space can be stated to be one reason for starting early interceptive treatment for crowding in the permanent dentition. Early interceptive space opening for permanent teeth by means of a Quad Helix [15], an eruption guidance appliance [16], or headgear [17–19] will effectively create the necessary space in the dental arch at the early mixed stage in the dentition. Thus, early dental headgear treatment has been shown to have a straightening effect on the eruption path of the maxillary canines [18, 19].

In the case of scarce interceptive treatment or the late diagnosis of a disturbance in the eruption of a canine, later orthodontic treatment, possibly including surgical treatment, would be needed. Late treatment for displaced canines is usually a long-term and expensive procedure [20] and is always a tougher treatment option for the child and the family.

Delayed dental age has been shown earlier to be associated with the impaction of a maxillary canine [21, 22] and has been connected in some studies with palatal displacement in particular [23, 24], but other authors have not reported any such findings [25]. In our earlier study, delayed dental age was associated with the early treatment of maxillary canines, including the extraction of a primary canine, using leeway space [26] by interceptive slicing of a primary second molar or early dental headgear treatment [27].

The aim of the present work was to describe differences in the dental age of Finnish children at the mixed stage of dentition in relation to the treatment provided for the erupting maxillary canines, the hypothesis being that dental age varies among children with different timing of the treatment for the maxillary canines. A second hypothesis was that advanced dental development occurred more often in children receiving late treatment for their maxillary canines.

## Materials and methods

### Material

The cohort for this register-based study consisted of children of Finnish ancestry born in a given municipality in Eastern Finland between 1980 and 1996. The cross-sectional part of the work included 1,454 digitally copied dental panoramic tomographs (DPTs) of the developing permanent dentition. The children were mainly in the third year of primary school at the time of the DPT, taken between the years 1987 and 2007 in response to referrals during annual oral examinations in order to examine the condition of their dental development. The DPTs were taken using Cranex DC 2 (Soredex) equipment up to 15 March 2006 and a Planmeca Proline XC (Plandent) system thereafter. Longitudinal information on the eruption of the maxillary canines and the treatment carried out for them was obtained by examining the dental records and other dental radiographs found in the paper archives or the electronic patient registration system. The data were gathered retrospectively from the archives of the municipal health center between the years 2006–2007 (DPTs) and 2016–2020 (longitudinal information).

### Ethical considerations

In accordance with Finnish legislation (Act 552/2019), the data for this research were gathered from dental records in the patient registration system with the permission of the administrator of the register. Personal information was coded for the analyses to prevent identification.

### Dental age

Dental age was analyzed from the DPTs by EM and JI, assisted by a senior orthodontist MK, who was acquainted with the method. Demirjian’s dental maturity method [28, 29] was used and assessed by reference to Finnish maturity curves [8]. The developmental stages of seven individual teeth in the mandibular left quadrant were evaluated, or if a left mandibular tooth was absent, its antimere was taken for assessment [29], as also if the developmental stage of an individual tooth deviated notably from the general line. The exclusion criteria for this variable were an absent tooth and no antimere or poor quality of the DPT. The examiners assessed 30 DPTs twice to assess the accuracy of the assessments. For the purposes of this study, the children’s dental ages were grouped into delayed (≤ –1 year), normal (> –1 and < +1), and advanced (≥ +1 year) relative to their chronological age.

### Treatment

Decisions regarding treatment had been taken upon clinical inspection, including palpation and space conditions, for example, and radiological inspection by DPT and after follow-up whenever needed. Information on the eruption and treatment of the maxillary canines was collected retrospectively by JR, KK, and RL from the time after the DPT until the maxillary canine had been caused to erupt, the orthodontic treatment required for its eruption having been carried out according to a treatment plan made by a senior orthodontist RV. The exclusion criteria for this variable were an emerged canine (based on the DPT and/or dental records), oligodontia (> six absent teeth), poor quality of the DPT, odontoma or a cyst in the maxillary canine area, or transposition of a maxillary canine with the first premolar. Orthodontic treatment carried out before the DPT was taken or after the maxillary canine had erupted was not included. The treatments were categorized as shown in Table 1.

**Table 1:** Categorization of maxillary canines according to their eruption and treatment.

Treatment	Definition
**No treatment**	No treatment for maxillary canines; follow-up might have been carried out.
**Early treatment**	
Interceptive treatment	Extraction of a primary canine due to the eruption of a maxillary canine and/or use of leeway space for the canine-premolar region by interceptive slicing of a primary second molar and simultaneous extraction of a primary first molar if necessary to provide extra space in the canine and premolar region.
Early headgear	Dental headgear applied with an Interlandi set-up during mixed dentition before or during eruption of the maxillary canines, possibly including other interceptive procedures.
**Late treatment**	
Orthodontic treatment	Mainly surgical exposure of a maxillary canine and traction with a buccal bow (TMA 0.016 × 0.018) from the transpalatal arch and later alignment with a fixed appliance. Sometimes, surgical exposure alone or including traction with a fixed appliance was enough.
Treatment for crowding	Extraction of a permanent first premolar and/or treatment with a fixed appliance for relieving severe crowding, especially for a maxillary canine. In some cases, the extracted tooth may be a permanent second premolar.

### Inclusion criteria

The inclusion criteria for the present study were chronological age 8.5–10.5 years at the time of the DPT, dentition in the mixed stage, and no syndromes or clefts.

### Statistics

The statistical analyses were performed using IBM SPSS Statistics (version 28.0) and SAS Enterprise guide 7.1. *P*-values < 0.05 were considered statistically significant. Repeatability of the dental age assessments was assured using intra-class correlation (ICC).

The normality of variables such as chronological and dental ages was assessed visually using histograms. Differences in mean chronological age between the genders were analyzed with the independent samples *t*-test, and differences in mean chronological ages between the treatment groups of maxillary canines were analyzed with generalized linear mixed-effect models with a random effect, to take account of children having two canines in the data. Statistically significant two-way interaction terms were checked during the formation of the models.

The distributions of the variables were described in terms of frequencies and percentages. The comparisons between the maxillary canine treatment groups and the dental age groups were performed using either Pearson’s chi-square test or Fisher’s exact test.

The individual associations of dental age (delayed, normal, advanced) with the response variables (maxillary canine treatment group) were determined using crude multinominal logistic regression models. The strength of the association being illustrated with an odds ratio (OR) and 95% confidence interval (95% CI). The multinominal logistic regression models were resolved using generalized linear mixed models with random effect, to take account of children having two canines in the data.

## Results

### Repeatability

The intra-rater repeatability for dental age was ICC = 0.789 for examiner 1 and ICC = 0.945 for examiner 2, while inter-rater reliability was ICC = 0.871. Thus, the assessments of dental age proved to be reliable in terms of their repeatability.

### Descriptive statistics

The material consisted of 1,332 DPTs for 627 girls and 705 boys with chronological ages in the range 8.5–10.5 years, their overall mean age at the time of the DPT being 9.4 years (standard deviation [SD] 0.4), a figure that did not differ between the genders (*p* = 0.052). Dental age could be assessed for 555 girls, with the mean of 9.6 years (SD 0.8) and a range between 6.5 and 13.2 years, and for 636 boys, with the mean of 9.8 years (SD 1.0) and a range between 7.0 and 12.7 years.

The treatment provided could be assessed for 431 girls and 500 boys, including a total of 1,817 maxillary canines. A total of 177 canines were actually treated (110 children), as most of them (90.3%, *n* = 1,640) did not need treatment. Early treatment was the option chosen for 90 canines (55 children), including 39 (30 children) managed with interceptive treatment and 51 canines (27 children) with early headgear. Late treatment was provided for 87 canines (58 children), including 16 (13 children) receiving orthodontic treatment and 71 canines (45 children) receiving treatment for crowding.

### Chronological and dental ages

The mean chronological and dental ages of the children concerned at the time of the DPT are presented by treatment groups and gender in Table 2. No differences (diff.) in mean chronological age were found between the treatment groups among the girls, but the mean dental age in the early treatment group was significantly delayed relative to the girls receiving no treatment (diff. 0.43 years, *p* = 0.004) or late treatment (diff. 0.45 years, *p* = 0.026). In the boys, the chronological age of the late treatment group at the time of the DPT was significantly lower than that of the group receiving no treatment (diff. 0.19 years, *p* = 0.002). No differences in mean dental age were found between the groups.

**Table 2:** Differences in the mean chronological and dental ages of the children by treatment groups.

	Mean chronological age in years (SD)	*P* ^a^	*P* ^b^	Mean dental age in years (SD)	*P* ^a^	*P* ^b^
**Girls**						
No treatment	9.37 (0.4)			9.60 (0.8)		
Early treatment	9.44 (0.3)	0.210	0.170	9.17 (0.9)	0.004	0.026
Late treatment	9.33 (0.4)	0.501		9.62 (0.6)	0.898	
**Boys**						
No treatment	9.46 (0.4)			9.95 (0.9)		
Early treatment	9.36 (0.4)	0.087	0.246	9.64 (1.0)	0.059	0.417
Late treatment	9.27 (0.4)	0.002		9.81 (1.1)	0.333	

a Linear mixed model, significances are of differences between no treatment and early or late treatment.

b Linear mixed model, significances are of differences between early and late treatment. SD: standard deviation.

### Dental age and treatment

Most of the children in each treatment group had a normal dental age at the time of the DPT (Table 3), but a delayed dental age was detected significantly more often in the early treatment group, whereas an advanced dental age was detected more often in the late treatment group (*p* = 0.002). The majority of the early-treated children with delayed dental age were in the interceptive treatment group, where almost a third of the children had a delayed dental age (*p* = 0.019). Among the late-treated children, a fifth of the children who received orthodontic treatment and more than a third of those treated for crowding showed advanced dental age (*p* = 0.806).

**Table 3:** Treatment of maxillary canines vs. dental age^a^ reported here, by individual maxillary canines.

	Delayed^b^		Normal^b^		Advanced^b^		*P*
	*n*	%	*n*	%	*n*	%	
**Treatment**							
No treatment	77	5.1	1,126	74.6	306	20.3	
Early treatment	11	15.1	57	78.1	5	6.8	0.002^c^
Late treatment	6	7.9	48	63.2	22	28.9	
Total	94		1,231		333		< 0.001^d^
**Treatment**							
Interceptive treatment	9	28.1	21	65.6	2	6.3	0.019^d,e^
Early headgear	2	4.9	36	87.8	3	7.3	
Orthodontic treatment	1	6.7	11	73.3	3	20.0	0.806^d,f^
Treatment for crowding	5	8.2	37	60.7	19	31.1	
Total	17		105		27		< 0.001^d^

a Dental age in children as assessed by Demirjian’s method [28, 29] was the same for both maxillary canines.

b Delayed (≤ –1 year), normal (> –1 and < +1 year), and advanced (≥ +1 year) relative to the chronological age.

c Pearson’s chi-square test, significance is of the difference between the early and late treatment groups.

d Fisher’s exact test.

e Significance is of the difference between the interceptive treatment and early headgear groups.

f Significance is of the difference between the orthodontic treatment and treatment for crowding groups.

The multinominal crude logistic regression analysis pointed to an association between delayed dental age and interceptive treatment of a maxillary canine (OR 3.99, 95% CI 1.84–8.67) (Table 4), no significant association was found between advanced dental age and late treatment of a maxillary canine.

**Table 4:** Crude multinominal logistic regression associations between the independent variable dental age^a^ (delayed vs. normal and advanced vs. normal) and the dichotomous dependent variables no treatment vs. maxillary canine treatment groups.

Treatment	Delayed		Advanced	
	OR	95% CI	OR	95% CI
**All treatments**	**2.57**	**1.15–5.76**	0.95	0.53–1.70
**Early treatment**	**2.96**	**1.14–7.69**	0.38	0.13–1.06
Interceptive treatment	**3.99**	**1.84–8.67**	0.75	0.32–1.75
Early headgear	0.92	0.26–3.21	0.62	0.28–1.38
**Late treatment**	1.81	0.60–5.50	1.69	0.88–3.23
Orthodontic treatment	1.43	0.16–12.75	1.10	0.28–4.25
Treatment for crowding	1.91	0.54–6.72	1.89	0.91–3.89

a Dental age in children as assessed by Demirjian’s method [28, 29] was the same for both maxillary canines, ref. normal. OR: odds ratio; CI: confidence interval. Statistically significant values (p < 0.05) are in bold.

## Discussion

The results describe the differences in dental age at the mixed stage of dentition in relation to the treatment provided for the erupting maxillary canines in a Finnish children age cohort 8.5–10.5 years. These offer a new perspective on permanent maxillary canine eruption problems relative to the treatment given in previous studies of the maxillary canines and dental age, which have focused on displaced or impacted canines [21–25].

The children who received interceptive treatment for their maxillary canine(s) significantly more often had a delayed dental age, as almost a third of this group did so. This result was confirmed in the logistic regression analysis, where delayed dental age was associated with interceptive treatment for a maxillary canine. On the other hand, delayed dental age was not significant for children treated with early headgear.

Properly timed extraction of the primary canine [11–13] is a widely used interceptive treatment option for a displaced permanent canine, since it reduces the prevalence of impactions [14]. We saw here that extraction of a primary canine and interceptive slicing of a primary second molar, with simultaneous extraction of a primary first molar, is needed for certain space conditions. This ‘usage of leeway space’ can preserve space in the canine-premolar region, thus preventing mesial drift of the first molar. However, it is important to bear in mind the use of dental headgear and/or a lingual bar whenever necessary in order to achieve or maintain a normal first molar occlusion.

The mean dental age of the girls was almost half a year lower in the group with early-treated canines than that in those receiving no treatment (–0.43 years) or late treatment (–0.45 years). It is also notable that the girls were congruent in terms of chronological age. Similar significant findings regarding dental age were not forthcoming in the boys, however. Here the chronological age in boys of the late treatment group was significantly lower than that of the no treatment group (–0.19 years), but the differences in dental age between the groups were not significant. Thus, the hypothesis of variation in dental age among the groups of children with different maxillary canine treatment groups studied here can be accepted.

Advanced dental age was seen more often in the late maxillary canine treatment groups than in the no treatment and early treatment groups. The child’s dental age was assessed as advanced in more than a third of the maxillary canines treated for crowding and in a fifth of those treated orthodontically. Advanced dental age was emphasized in the treatment for the crowding group, which underlines the importance of evaluating space conditions relative to the stage of dental development. Labial positioning of the canine is usually treated by gaining more space in the dental arch, and for this purpose, the dental headgear gives extra space and correct or maintain the first molar occlusion favorably when dental development is approaching the second mixed stage. The hypothesis that advanced dental development occurred more often in children with late treatment of their maxillary canines was supported by the present results, but advanced dental age was not associated with late treatment of a maxillary canine.

Some of the children had received orthodontic treatment before the DPT was taken (for the primary dentition or during the early mixed stage), e.g. in the form of a Quad Helix for posterior cross-bite, braces on the upper incisors to close a large medial diastema, slicing of the primary canines mesially in the case of minimum space deficiency during the eruption of the lateral incisors, or elastics for a first molar cross-bite, which can be considered a limitation of this study. Due to the retrospective approach adopted here, it is good to understand that the timing of individual treatments had been based on demand and determined at yearly oral check-ups or half-yearly follow-ups after DPT.

Delayed dental age appeared four times more often (OR 3.99, 95% CI 1.84–8.67) in the children whose maxillary canines had been treated only by the extraction of the primary canine and/or usage of leeway space. When dental development is delayed and the eruption of the canine has been found to be aberrant, it is good to monitor the eruption until the second mixed stage before reaching the eventual treatment decision. Monitoring means inspection and palpation every half year and intraoral radiography when needed. The space conditions need to be assessed, as does the clinical eruption of the other permanent teeth. In order to continue the follow-up, the position of the canine in relation to the adjacent teeth will need to be improved. Each individual eruption sequence can be found in the DPT, and each should play its part in affecting the decision to monitor or treat.

The timing of treatment plays a key role in guiding the eruption of the maxillary canines. If early treatment does not eliminate the eruption problem, it will still reduce the treatment time and the degree of difficulty of any later treatment. The result obtained with this age cohort of 8.5–10.5 years indicates that the children who had a delayed dental age more often needed early interceptive treatment, whereas an advanced dental age was more commonly detected in the children whose canine(s) required treatment for crowding. Estimation of the dental development stage of each individual is crucial, and attention should be paid to space conditions after the first mixed stage in order to consider the early opening up of space for the permanent teeth and the erupting maxillary canines.

## Conclusions

In this age cohort (8.5–10.5 years):

- Normal dental age was presented in all studied treatment groups for the most part.
- Delayed dental age was detected more often in the early treatment group and advanced dental age in the late treatment group.
- The mean dental age of the girls in the early treatment group was delayed by almost half a year compared with the girls in the no treatment and late treatment groups.
- Most of the early-treated canines were in the interceptive treatment group (extraction of a primary canine and/or using leeway space by interceptive slicing of a primary second molar and simultaneous extraction of a primary first molar if needed), where almost a third of the children had a delayed dental age.
- Delayed dental age was associated with interceptive treatment of a maxillary canine.
- The timing of treatment is crucial to achieve early treatment options for children’s erupting maxillary canines due to a variable dental age within the same age group.
